# Regional Policies Targeting Residential Solid Fuel and Agricultural Emissions Can Improve Air Quality and Public Health in the Greater Bay Area and Across China

**DOI:** 10.1029/2020GH000341

**Published:** 2021-04-01

**Authors:** Luke Conibear, Carly L. Reddington, Ben J. Silver, Christoph Knote, Stephen R. Arnold, Dominick V. Spracklen

**Affiliations:** ^1^ Institute for Climate and Atmospheric Science School of Earth and Environment University of Leeds Leeds UK; ^2^ Faculty of Medicine University of Augsburg Germany

**Keywords:** ambient air pollution, China, greater bay area, health impact assessment, particulate matter, policy scenario

## Abstract

Air pollution exposure is a leading public health problem in China. The majority of the total air pollution disease burden is from fine particulate matter (PM_2.5_) exposure, with smaller contributions from ozone (O_3_) exposure. Recent emission reductions have reduced PM_2.5_ exposure. However, levels of exposure and the associated risk remain high, some pollutant emissions have increased, and some sectors lack effective emission control measures. We quantified the potential impacts of relevant policy scenarios on ambient air quality and public health across China. We show that PM_2.5_ exposure inside the Greater Bay Area (GBA) is strongly controlled by emissions outside the GBA. We find that reductions in residential solid fuel use and agricultural fertilizer emissions result in the greatest reductions in PM_2.5_ exposure and the largest health benefits. A 50% transition from residential solid fuel use to liquefied petroleum gas outside the GBA reduced PM_2.5_ exposure by 15% in China and 3% within the GBA, and avoided 191,400 premature deaths each year across China. Reducing agricultural fertilizer emissions of ammonia by 30% outside the GBA reduced PM_2.5_ exposure by 4% in China and 3% in the GBA, avoiding 56,500 annual premature deaths across China. Our simulations suggest that reducing residential solid fuel or industrial emissions will reduce both PM_2.5_ and O_3_ exposure, whereas other policies may increase O_3_ exposure. Improving particulate air quality inside the GBA will require consideration of residential solid fuel and agricultural sectors, which currently lack targeted policies, and regional cooperation both inside and outside the GBA.

## Key Points

- Ambient fine particulate matter exposure inside the Greater Bay Area is strongly controlled by emissions outside the Greater Bay Area
- Residential solid fuel and agricultural emissions lack effective controls that could improve air quality and public health across China
- Improving particulate air quality inside the Greater Bay Area will require regional cooperation inside and outside the Greater Bay Area

## 1 Introduction

Air pollution exposure was the fourth leading risk factor to the disease burden in China in 2017, associated with 7.5% (95% uncertainty interval, UI: 6.5–8.6) of the healthy life lost (GBD 2017 Risk Factor Collaborators, 2018; Yin et al., 2020). The majority of this disease burden was attributed to ambient fine particulate matter (PM_2.5_) exposure (71%), with contributions from household PM_2.5_ (23%) and ambient ozone (O_3_, 6%) exposure (GBD 2017 Risk Factor Collaborators, 2018). The health effects incurred by air pollution in the Pearl River Delta (PRD) in South China have been estimated to result in economic losses equivalent of up to 2.3% of regional gross domestic product (GDP, D. Huang et al., 2012; Lu et al., 2016). In the absence of air pollution controls, GDP losses at the provincial level may exceed 3% by 2030 (Xie et al., 2016). This highlights the need for carefully designed policies that will reduce air pollution.

There was a 5% reduction in the loss of healthy life associated with air pollution exposure from 2013 to 2017 (8.0%, 95UI: 6.9 to 9.0, GBD 2017 Risk Factor Collaborators, 2018). This improvement in public health was primarily attributed to reduced PM_2.5_ exposure, resulting from emission reductions of the 2013–2017 Air Pollution Prevention and Control Action Plan (APPCAP, Cheng et al., 2019; Ding et al., 2019; Guo et al., 2018; J. Huang et al., 2018; X. Jiang et al., 2015; Li et al., 2019a Ministry of Environmental Protection of China, 2013; Silver et al., 2020a; B. Zheng et al., 2018; Y. Zheng et al., 2017b). For example, a key region targeted by the APPCAP was the PRD which achieved a 28% reduction in ambient PM_2.5_ concentrations (from 47 μg m^−3^ to 34 μg m^−3^), exceeding the targeted 15% reduction (China Ministry of Ecological Environment, 2013, 2017). This reduction in ambient PM_2.5_ concentrations was primarily due to emission reductions in the industrial and power generation sectors (Ding et al., 2019; B. Zheng et al., 2018; Q. Zhang et al., 2019).

Previous work on air pollution in the PRD has found that emissions outside the PRD contributed 53% and local emissions contributed 47% to ambient PM_2.5_ concentrations inside the PRD for 2010 (Hou et al., 2019). The contribution of emissions outside the PRD to ambient PM_2.5_ concentrations inside the PRD was highest in winter and autumn, likely due to the strong prevailing north‐easterly wind (Hou et al., 2019). X. Jiang et al., (2015) found that ambient PM_2.5_ concentrations across the PRD in 2017 were 20% primary (organic and black carbon), 45% secondary (sulfate, nitrate, and ammonium), with 35% from other components. Fang et al., (2019) found that air quality in the Guangdong‐Hong Kong‐Macau Greater Bay Area (GBA) over 2015–2017 was worst in Foshan, Guangzhou, and Dongguan, likely due to both large local emissions and accumulation of secondary pollution. Ambient PM_2.5_ concentrations were lower in cities near the Pearl River estuary, such as Hong Kong and Shenzhen, likely due to more effective dilution by the cleaner surrounding marine air (Fang et al., 2019). Industrial emissions dominated the contribution to ambient PM_2.5_ concentrations in the PRD in 2010 (Reddington et al., 2019). Emissions across the PRD have changed in recent years. From 2010 to 2015, sulfur dioxide (SO_2_) emissions reduced by 43%, PM_2.5_ emissions reduced by 28%, and nitrogen oxides (NO_X_) emissions reduced by 13%, likely due to control measures on industry and energy generation (Bian et al., 2019; B. Zheng et al., 2018). In contrast, emissions of volatile organic compounds (VOCs) increased by 11% in the PRD over 2010–2015 (Bian et al., 2019; B. Zheng et al., 2018). Much of the PRD is VOC‐limited, especially in autumn and winter, suggesting that increasing VOC emissions and decreasing NO_X_ emissions may increase O_3_ concentrations (Jin & Holloway, 2015; Ou et al., 2016; Wang et al., 2017). Emissions of ammonia (NH_3_), dominated by the agricultural sector, stayed approximately the same (Bian et al., 2019). This highlights the difficulty in simultaneously reducing local PM_2.5_ and O_3_ concentrations (H. Liu et al., 2013). Indeed, long−term monitoring studies show PM_2.5_ concentrations across the PRD and GBA have declined in recent years, whereas O_3_ concentrations have increased (Silver et al., 2020a; Silver et al., 2018). Despite improvements, PM_2.5_ concentrations across the GBA are still damaging to public health. Epidemiological studies of air pollution exposure in the GBA find associations with mortality and morbidity (Hedley et al., 2002; Peters et al., 1996; Y. Tao et al., 2012; Z. Zhang et al., 2014), in addition to the epidemiological studies across the whole of China (Baumgartner et al., 2014; R. Chen et al., 2019; Clark et al., 2019; Ebenstein et al., 2017; T. Li et al., 2018; F. Liang et al., 2019a; L. Liang et al., 2019b; Rich et al., 2015; Y. Tao et al., 2012; Tian et al., 2019; Wu et al., 2018; Yan et al., 2019; Yin et al., 2017).

Despite these recent improvements in air quality, ambient PM_2.5_ concentrations across China still largely exceed the national standard (35 μg m^−3^) and ambient O_3_ concentrations increased (Silver et al., 2018; Silver et al., 2020b). There is a lack of effective emission control measures for the residential and agriculture sectors, which are both key contributors to the associated disease burden (Cai et al., 2018; Reddington et al., 2019; H. Zheng et al., 2019). The national 2018–2020 3‐year plan requires emission reductions of 15% in SO_2_, 15% in NO_*X*_, and 10% in VOCs. The 3‐year plan also requires that all cities that exceeded the annual‐mean ambient PM_2.5_ concentration standard of 35 μg m^−3^ in 2015 to achieve 18% reductions by 2020 (Ministry of Environmental Protection of China, 2018).

We used a regional chemical transport model to explore how potential air quality policy scenarios can address these issues surrounding ambient air pollution exposure and the loss of healthy life across China, with a specific focus on the GBA. We explored the impacts of a range of potential policies and contrasted emission sectors where existing policies are already being implemented (such as land transport and industry) to sectors lacking effective policies (such as residential and agriculture).

## 2 Methods

### 2.1 Model

Simulations were conducted using the Weather Research and Forecasting model online‐coupled with Chemistry (WRFChem) version 3.7.1 (Grell et al., 2005; Skamarock et al., 2008). Detailed information on the model setup is provided in Table S1 and in our previous work (Reddington et al., 2019; Silver et al., 2020a). The model domain covered China at 30 km (∼0.3°) horizontal resolution with a 10 km (∼0.1°) nest over the GBA (Figure S1). The GBA is an urban area surrounded by rural areas, and includes Dongguan, Foshan, Guangzhou, Huizhou, Jiangmen, Shenzhen, Zhaoqing, Zhongshan, Zhuhai, Hong Kong, and Macao.

Anthropogenic emissions for China were provided by the Multi‐resolution Emission Inventory for China (MEIC) emission inventory at 0.25 × 0.25° horizontal resolution (M. Li et al., 2017; MEIC Research Group & Tsinghua University, 2019; B. Zheng et al., 2018). Emissions were for black carbon, organic carbon, PM_2.5_, coarse particulate matter (PM_10_), carbon monoxide, NH_3_, NO_*X*_, SO_2_, and non‐methane VOCs. VOCs were speciated according to the Model for Ozone and Related Chemical Tracers (MOZART, Emmons et al., 2010). Anthropogenic emissions of methane inside China, and all anthropogenic emissions outside of China, were from the Emission Database for Global Atmospheric Research with Task Force on Hemispheric Transport of Air Pollution (EDGAR‐HTAP) version 2.2 for 2010 at 0.1 × 0.1° horizontal resolution (Janssens‐Maenhout et al., 2015). Sectoral emissions were provided for land transport, industry, residential energy use, power generation, shipping, aircraft, and agriculture. A diurnal cycle was applied to the anthropogenic emissions (Qi et al., 2017; B. Zheng et al., 2017a).

Open biomass burning emissions were from the Fire Inventory from National Center for Atmospheric Research (FINN) version 1.5 (Wiedinmyer et al., 2011), with emissions distributed evenly throughout the boundary layer. Biogenic emissions were calculated online using the Model of Emissions of Gases and Aerosol from Nature (MEGAN, Guenther et al., 2006). Dust emissions were calculated online using Global Ozone Chemistry Aerosol Radiation and Transport (GOCART) with Air Force Weather Agency modifications (Legrand et al., 2019).

Gas phase chemistry was simulated using the extended MOZART scheme (Emmons et al., 2010; A. Hodzic & Jimenez, 2011; Knote et al., 2014). Aerosol physics and chemistry were simulated using the updated Model for Simulating Aerosol Interactions and Chemistry (MOSAIC) scheme, with aqueous chemistry and four sectional discrete size bins; 0.039–0.156 μm, 0.156–0.625 μm, 0.625–2.5 μm, and 2.5–10 μm (Alma Hodzic & Knote, 2014; Zaveri et al., 2008). The secondary organic aerosol formation was based on an updated volatility basis set mechanism (Knote et al., 2015).

Microphysics were simulated using the Morrison two‐moment scheme (Morrison et al., 2009). Chemical initial‐ and boundary‐conditions were taken from the MOZART/Goddard Earth Observing System Model (National Center for Atmospheric Research 2016). Meteorological initial‐ and boundary‐conditions were taken from the European Center for Medium‐Range Weather Forecasts Re‐Analysis (ERA)‐Interim global product (Dee et al., 2011), on a N256 (∼35 km at the equator) grid at the surface, on a N128 (∼70 km at the equator) grid above the surface, and updated every 6 h. WRF meteorology was nudged to these fields above the boundary layer.

### 2.2 Simulations

We performed two sets of simulations. The first set of simulations were to explore the relative roles of emissions inside and outside the GBA on ambient air pollution within the GBA. The second set of simulations explored the impacts of specific policy scenarios implemented inside and outside the GBA on ambient air quality and human health inside the GBA and across China.

The first set of simulations were for the months of April 2014, July 2014, October 2014, and January 2015, using anthropogenic emissions from MEIC for the corresponding years (2014 and 2015) with offline‐nesting. The outer domain of each simulation was spun‐up for 1 month, and the inner domain was spun‐up for 3 days. A control simulation was performed with no changes in anthropogenic emissions. Simulations were implemented with 15% reductions in all anthropogenic emissions inside and outside the GBA. The 15% emission reduction was chosen as it is the average emission reduction target for Hong Kong and the PRD region in 2020 relative to 2015 across SO_2_, NO_*X*_, PM_10_, and VOC emissions (Hong Kong Environment Bureau & Ministry of Environmental Protection of China, 2019).

The second set of simulations were for the year of 2015, each with a 1−month spin−up, using anthropogenic emissions from MEIC for 2015 with online‐nesting. A control (CTL) scenario was simulated where anthropogenic emissions were kept at 2015 levels. Six simulations were performed to represent relevant air quality policy scenarios (Table 1).

**Table 1:** *Air Quality Policy Scenarios*

Scenario	Description
**CTL**	A control scenario for 2015 in China
**RES**	Reduce residential emissions by 50% outside the GBA to approximate a 50% transition to liquefied petroleum gas
**IND**‐**GBA**	Reduce industrial volatile organic compound emissions by 10% inside the GBA to approximate the attainment of this aim from the 2018–2020 3‐year plan
**IND**‐**CHN**	Same as for IND‐GBA, though applied to the whole of China
**TRA**‐**GBA**	Reduce land transport nitrogen oxides emissions by 80% inside the GBA to approximate the aim of the 2018–2020 3‐year plan to enhance land transport standards in the Pearl River Delta through transitioning away from diesel
**TRA**‐**CHN**	Same as for TRA‐GBA, though applied to the whole of China
**AGR**	Reduce agricultural ammonia emissions by 30% outside the GBA to approximate the attainment of this aim from the 2018–2020 3‐year plan

*Note*. Emission changes were applied to the residential (RES), industrial (IND), land transport (TRA), and agricultural (AGR) sectors, relative to a control (CTL), either inside the Guangdong‐Hong Kong‐Macau Greater Bay Area (GBA) or across China (CHN).

Two scenarios focused on the industrial sector, as this is a key source of VOC emissions in China, which are important precursors to ambient O_3_ concentrations. We reduced industrial VOC emissions by 10% in accordance with the aims of the 2018–2020 3‐year plan, separately inside (IND‐GBA) and outside the GBA (IND‐CHN).

Two scenarios focused on the land transport sector, as this is a key source of NO_*X*_ emissions in China, which are another important precursor to ambient O_3_ concentrations. The 2018–2020 3‐year plan requires the PRD to enhance land transport standards and transition away from diesel to decrease NO_*X*_ emissions. We reduced land transport NO_X_ emissions by 80% to approximate diesel vehicles implementing the China six emission standard (X. Liang et al., 2019c). We applied this separately inside (TRA‐GBA) and outside the GBA (TRA‐CHN).

One scenario focused on the residential sector, as this is a leading contributing sector to air quality degradation across China and lacks effective emission controls (Reddington et al., 2019; Zhao et al., 2018; H. Zheng et al., 2019). The 2018–2020 3‐year plan introduced specific policies for the residential sector in Beijing‐Tianjin‐Hebei and the surrounding areas in winter to achieve 70% clean heating by 2021 under the Clean Heating Plan (Ministry of Environmental Protection of China, 2017; National Development and Reform Commission of China, 2017). However, there are no specific policies for reducing the use of solid fuels for residential cooking and heating in South China. Solid fuels accounted for 80% of the total fuel consumption by energy in rural households in the PRD in 2017 (X. Jiang et al., 2015). We simulated a 50% transition from solid fuels to liquefied petroleum gas (LPG) outside the GBA by reducing residential emissions from solid fuel use by 50% (RES) as being representative of a partial transition under continued fuel stacking (Barrington‐Leigh et al., 2019; Carter et al., 2019; S. Tao et al., 2018; Zhu et al., 2018).

One scenario focused on the agricultural sector, as this important sector has previously lacked effective emission controls (Cai et al., 2018; H. Zheng et al., 2019). The 2018–2020 3‐year plan required agricultural NH_3_ emissions to reduce by 40% in Beijing‐Tianjin‐Hebei and Yangtze River Delta by 2020. Previous work has suggested national use of fertilizer could be reduced by 30% whilst maintaining crop yields (X. Chen et al., 2014; X. Liu et al., 2016). Here we simulated the more conservative 30% reduction in agricultural NH_3_ emissions nationally outside the GBA (AGR).

The agricultural and residential scenarios are intended to be approximations of expanding realistic policies at the regional scale. They are not intended to be simulations of the exact policies under the more spatially limited measures in the 2018–2020 3‐year plan and clean heating plan, respectively.

### 2.3 Model Evaluation

**Figure 1:**
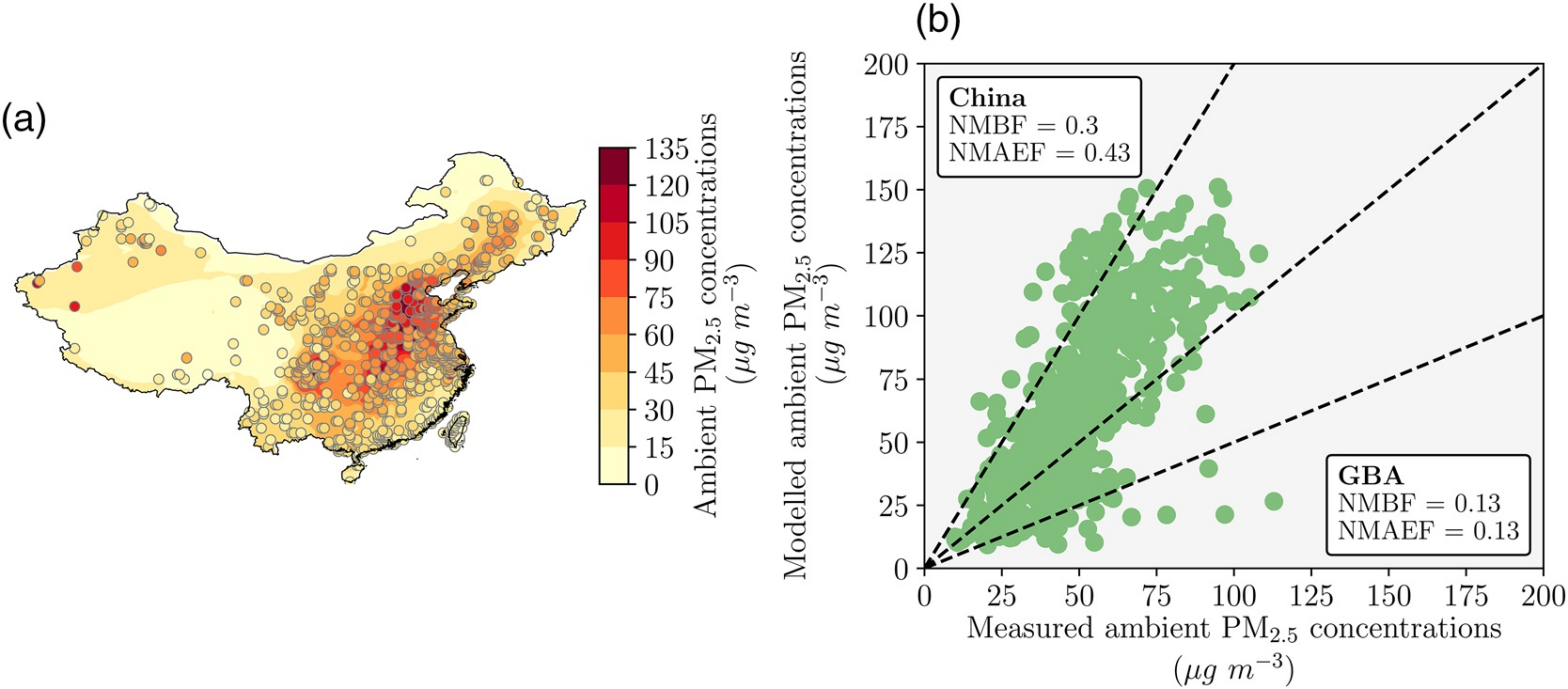
Evaluation of modeled ambient fine particulate matter (PM_2.5_) concentrations against measurements for 2015. (a) Simulated (background, combined domain) and measured (circles) annual‐mean PM_2.5_ concentrations. (b) Simulated (combined domain) versus measured annual−mean PM_2.5_ concentrations across China and inside the Guangdong‐Hong Kong‐Macau Greater Bay Area (GBA). Evaluation metrics were the normalized mean bias factor (NMBF) and the normalized mean absolute error factor (NMAEF). Across China, the NMBF was 0.30 and the NMAEF was 0.43. Inside the GBA, the NMBF was 0.13 and the NMAEF was 0.13. Dotted lines show the 1:1, 2:1, and 1:2 ratios.

**Figure 2:**
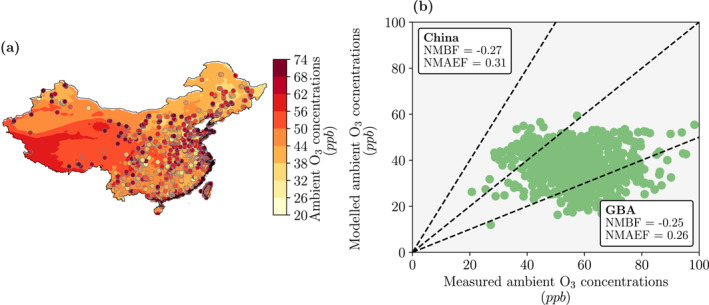
Evaluation of modeled ambient ozone (O_3_) concentrations against measurements for 2015. (a) Simulated (background, combined domain) and measured (circles) annual‐mean O_3_ concentrations. (b) Simulated (combined domain) versus measured annual‐mean O_3_ concentrations across China and inside the Guangdong‐Hong Kong‐Macau Greater Bay Area (GBA). Evaluation metrics were the normalized mean bias factor (NMBF) and the normalized mean absolute error factor (NMAEF). Across China, the NMBF was −0.27 and the NMAEF was 0.31. Inside the GBA, the NMBF was −0.25 and the NMAEF was 0.26. Dotted lines show the 1:1, 2:1, and 1:2 ratios.

We compared simulated ambient PM_2.5_ (Figure 1) and O_3_ concentrations (Figure 2) against hourly measured data from over 1,600 sites across China, Macao, Hong Kong, and Taiwan as detailed in Silver et al. (2018). The same model setup was evaluated in our previous work (Reddington et al., 2019; Silver et al., 2020a). For the first set of simulations in 2014, we evaluated PM_2.5_ concentrations from the nested domain over the GBA. For the second set of simulations in 2015, we evaluated a combined domain that overlaid the nested domain on top of the parent domain. The normalized mean bias factor (NMBF) and the normalized mean absolute error factor (NMAEF) were used to evaluate the model (S. Yu et al., 2006).

For the first set of simulations, the model slightly overestimated PM_2.5_ concentrations in October 2014 (NMBF = 0.09 and NMAEF = 0.13), July 2014 (NMBF = 0.24 and NMAEF = 0.28), and January 2015 (NMBF = 0.26 and NMAEF = 0.26). The model slightly underestimated PM_2.5_ concentrations in April 2014 (NMBF = −0.24 and NMAEF = 0.28).

For the second set of simulations, the model slightly overestimated PM_2.5_ concentrations (NMBF = 0.30 and NMAEF = 0.43, Figure 1) and slightly underestimated O_3_ concentrations (NMBF = −0.27 and NMAEF = 0.31, Figure 2) across China. The model biases were smaller over the GBA for both PM_2.5_ (NMBF = 0.13 and NMAEF = 0.13) and O_3_ (NMBF = −0.25 and NMAEF = 0.26) concentrations.

### 2.4 Health Impact Assessment

The health impact assessment used the second set of simulations for 2015 to estimate the disease burden attributable to air pollution exposure using population attributable fractions (PAF) of relative risk (RR). Intervention−driven variations in exposure were used to predict associated variations in outcome.

The exposure to PM_2.5_ (z) per grid cell was relative to the counterfactual exposure level of 2.4 μg m^−3^ (cf) where no excess risk was assumed (Equation 1). The RR for a specific exposure and population age group was estimated through the Global Exposure Mortality Model (GEMM, Burnett et al., 2018). The RR was a function of the parameters *θ*, *α*, *μ*, and *ν* (Equation 2, Table S2). We used the GEMM for non‐accidental mortality (non‐communicable disease, NCD, plus lower respiratory infections, LRI), using parameters that included the China cohort, with age‐specific modifiers for adults over 25 years of age in 5‐year intervals. The GEMM functions have mean, lower, and upper uncertainty intervals. The PAF was estimated as a function of the RR and the population count (P) (Equation 3)

(1) $$z=\max\left(0,\;PM_{2.5}-cf\right)$$

(2) $$RR\left(z,age\right)=e^{\left\{\theta \frac{\log\left(1+\frac{z}{\alpha }\right)}{1+e^{\left(\frac{\mu -z}{\nu }\right)}}\right\}}$$

(3) $$PAF=P\times \left(1-\frac{1}{RR\left(z,age\right)}\right)$$

The health impact assessment for O_3_ exposure followed the methodology of the Global Burden of Diseases, Injuries, and Risk Factors Study (GBD) for 2017 (GBD 2017 Risk Factor Collaborators, 2018). The exposure to O_3_ (z) per grid cell was calculated as the change in maximum 6‐monthly‐mean daily‐maximum 8‐hour O_3_ concentrations (6mDM8h), relative to the counterfactual exposure level of 35.7 ppb (cf) where no excess risk was assumed (Equation 4, Turner et al., 2016). The 6mDM8h was calculated by first quantifying 24 separate 8‐h rolling mean O_3_ concentrations, then finding the maximum of these each day, then creating 12 separate 6‐monthly means to account for seasonal variations, then finding the maximum of these over the year. The PAF was a function of the hazard ratio (HR), which was 1.06 (95UI: 1.02 to 1.10) for chronic obstructive pulmonary disease (COPD), based on data from five epidemiological cohorts (Equation 5, GBD 2017 Risk Factor Collaborators 2018).

(4) $$z=\max\left(0,\;O_{3}-cf\right)$$

(5) $$PAF=P\times \left(1-e^{z\frac{\log\;HR}{10}}\right)$$

Premature mortality (MORT), years of life lost (YLL), and years lived with disability (YLD) per exposure, health outcome, age bracket, and grid cell were estimated as a function of the PAF and the corresponding baseline mortality and morbidity rate (I_MORT_, I_YLL_, and I_YLD_) following Equations (6), (7), (8), respectively. Disability‐adjusted life years (DALYs), i.e. the total loss of healthy life, were estimated as the total of YLL and YLD following Equation 9. The rates of MORT, YLL, YLD, and DALYs were calculated per 100,000 population:

(6) $$MORT=PAF\times I_{MORT}$$

(7) $$YLL=PAF\times I_{YLL}$$

(8) $$YLD=PAF\times I_{YLD}$$

(9) DALYs=YLL+YLD

The United Nations adjusted population count dataset for 2015 at 0.05 × 0.05° resolution was obtained from the Gridded Population of the World, Version 4 (GPWv4) (Center for International Earth Science Information Network & NASA Socioeconomic Data and Applications Center, 2016). Population age composition was taken from the GBD2017 for 2015 for adults 25–80 years in 5‐year intervals, and for 80 years plus (Global Burden of Disease Study 2017, 2018). Cause‐specific (NCD, LRI, and COPD) baseline mortality and morbidity rates were taken from the GBD2017 for 2015 for MORT, YLL, and YLD for each age bracket (Institute for Health Metrics and Evaluation, 2020).

Shapefiles were used to aggregate results at the country, province, and prefecture level (Hijmans et al., 2016). Regional groupings were also applied as follows (Figure S2): North China (Beijing, Tianjin, Hebei, Shanxi, and Inner Mongolia), North East China (Liaoning, Jilin, and Heilongjiang), East China (Shanghai, Jiangsu, Zhejiang, Anhui, Fujian, Jiangxi, and Shandong), South Central China (Henan, Hubei, Hunan, Guangdong, Guangxi, Hainan, Hong Kong, and Macau) including the GBA, South West China (Chongqing, Sichuan, Guizhou, Yunnan, and Tibet), and North West China (Shaanxi, Gansu, Qinghai, Ningxia, and Xinjiang), and the GBA individually.

Uncertainty intervals at the 95% confidence level (95UI) were estimated through using the derived uncertainty intervals from the exposure−outcome associations, baseline mortality and morbidity rates, and population age fractions. Health impact assessments of the disease burden associated with air pollution exposure have many uncertainties, as detailed in Nethery & Dominici, (2019).

## 3 Results

### 3.1 Contribution of Emissions outside the GBA to Ambient Air Quality inside the GBA

**Figure 3:**
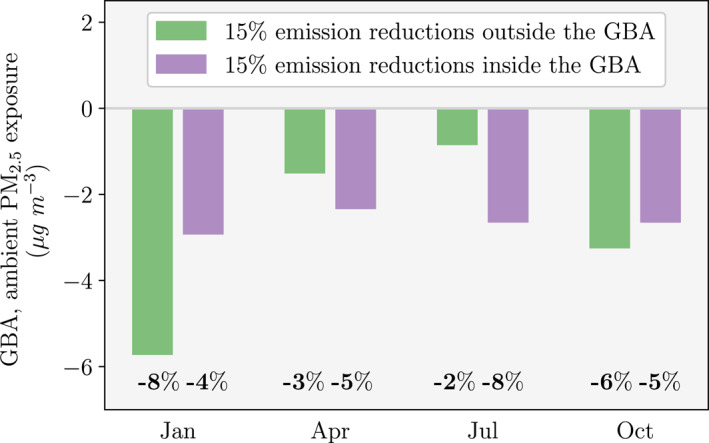
The impact of 15% emission reductions outside and inside the Guangdong‐Hong Kong‐Macau Greater Bay Area (GBA) on monthly–mean (January, April, July, and October) ambient fine particulate matter (PM_2.5_) exposure within the GBA. Simulated changes in PM_2.5_ exposure are from the nested domain at 10 km over the GBA.

Figure 3 compares the impacts of 15% emission reductions inside and outside the GBA on ambient PM_2.5_ exposure inside the GBA. Emission reductions outside the GBA produce larger reductions in PM_2.5_ exposure for January (8%) and October (6%) than emission reductions inside the GBA (4% for January and 5% for October). In contrast, emission reductions outside the GBA produce smaller reductions in PM_2.5_ exposure for April (3%) and July (2%) than emission reductions inside the GBA (5% for April and 8% for July). These results demonstrate the importance of pollution transport into the GBA in autumn and winter when ambient PM_2.5_ concentrations are generally higher than in spring and summer.

### 3.2 Current Disease Burden Associated with Ambient Air Pollution Exposure

**Figure 4:**
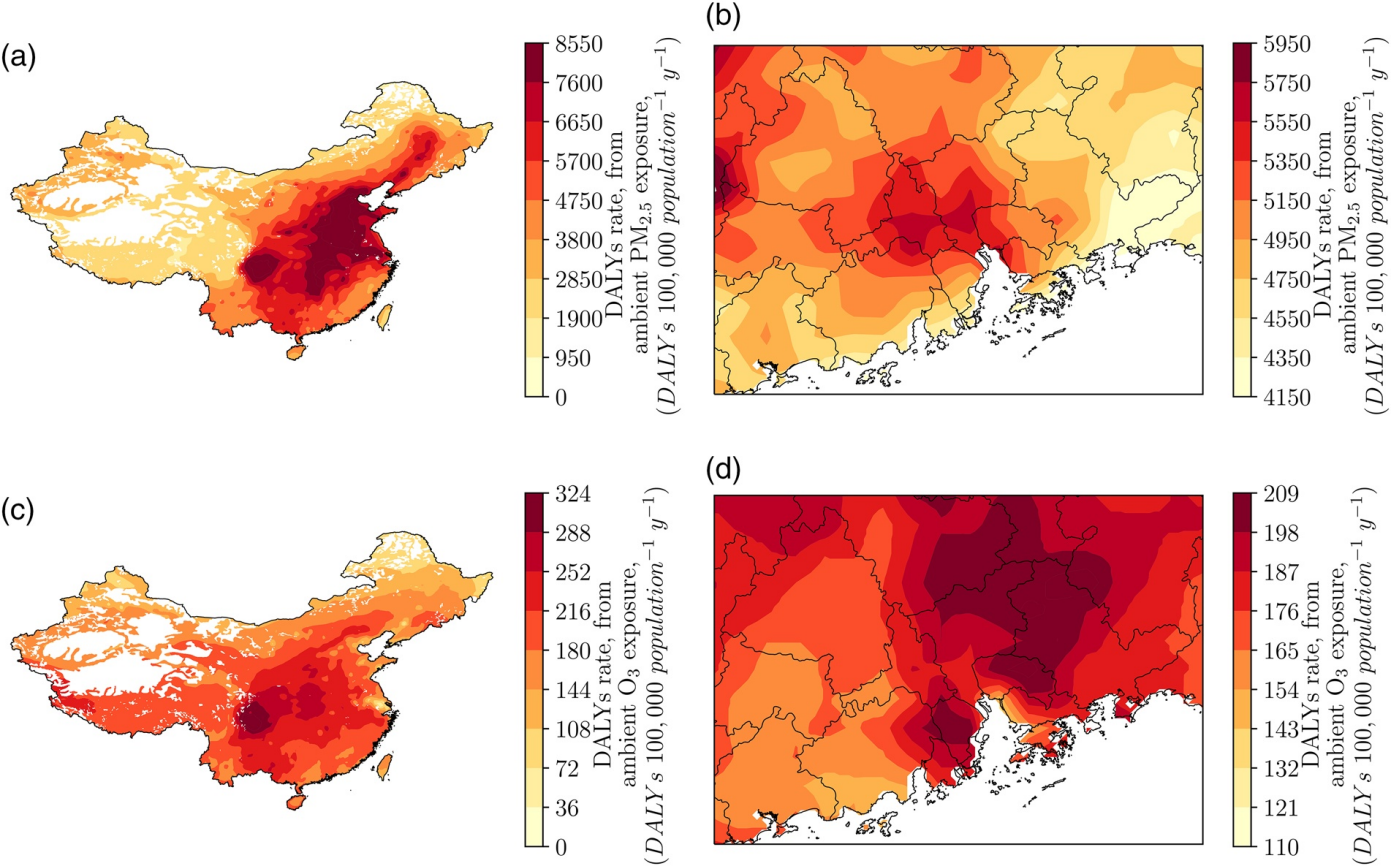
The disease burden associated with ambient air pollution exposure from the control scenario. Rate of disability‐adjusted life years (DALYs) per 100,000 population from (a) ambient fine particulate matter (PM_2.5_) exposure in China, (b) ambient PM_2.5_ exposure in the Guangdong−Hong Kong−Macau Greater Bay Area (GBA), (c) ambient ozone (O_3_) exposure in China, and (d) ambient O_3_ exposure in the GBA.

Figure 4 shows the simulated disease burden due to ambient PM_2.5_ and O_3_ exposure. The simulated population‐weighted annual‐mean PM_2.5_ exposure is 72.8 μg m^−3^ across China and 39.6 μg m^−3^ inside the GBA. The population‐weighted 6mDM8h O_3_ exposure is 63.5 ppb across China and 61.3 ppb inside the GBA. There are 2,779,000 (95UI: 2,701,000–2,865,000) premature deaths per year associated with ambient PM_2.5_ exposure in China, of which 105,000 (95UI: 102,000–108,000) premature deaths are inside the GBA. The PM_2.5_ disease burden is particularly large across East and South China. There are 123,000 (95UI: 86,000–171,000) premature deaths per year associated with ambient O_3_ exposure in China, of which 5,700 (95UI: 4,000–7,900) premature deaths are inside the GBA. The O_3_ disease burden is largest in South China. The DALYs rate from PM_2.5_ exposure is 4,476 (95UI: 3,947–5,084) per 100,000 population across China, and 4,887 (95UI: 4,309–5,551) per 100,000 population inside the GBA. The DALYs rate from O_3_ exposure is 186 (95UI: 122–267) per 100,000 population across China, and 182 (95UI: 120–262) per 100,000 population inside the GBA.

Our disease burden estimates are comparable to previous work. Our calculated disease burden associated with ambient PM_2.5_ exposure in China is 13% higher than that from Burnett et al. (2018), due to our higher estimate of PM_2.5_ exposure. Our calculated disease burden associated with ambient O_3_ exposure in China is 27% lower than that from the GBD 2017 Risk Factor Collaborators, (2018), due to our lower estimate of O_3_ exposure. Combined with our evaluation of PM_2.5_ and O_3_ concentrations, this demonstrates that our modeling framework is suitable for the assessment of emission scenarios on air quality and public health.

### 3.3 Impacts of Policy Scenarios on Ambient Air Quality and Public Health in China

**Figure 5:**
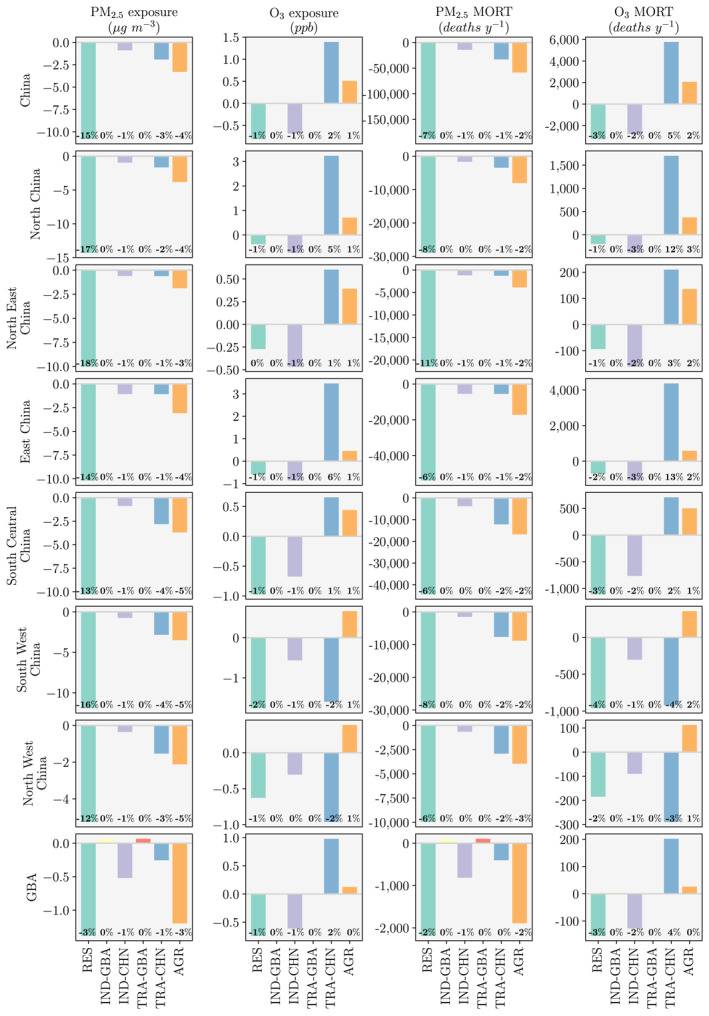
The impacts of policy scenarios on air quality and public health per region in China. Columns are population‐weighted annual−mean ambient fine particulate matter (PM_2.5_) exposure, population‐weighted maximum 6‐monthly‐mean daily‐maximum 8‐h ambient ozone (O_3_) exposure, and the annual‐sum of premature mortality (MORT) associated with PM_2.5_ and O_3_ exposure. Rows are for China, North China, North East China, East China, South Central China, South West China, North West China, and the Guangdong‐Hong Kong‐Macau Greater Bay Area (GBA, see Figure S2 for more details). Scenarios are for the residential (RES), industrial within the GBA (IND−GBA), industrial over China (IND−CHN), land transport within the GBA (TRA−GBA), land transport over China (TRA−CHN), and agriculture (AGR), with the percentage change relative to the control (see Table 1 for more details).

Figure 5 summarizes the impacts of each scenario on ambient air quality and public health. The results per region are in Table S3. Ambient air quality is reported as the population‐weighted annual‐mean PM_2.5_ exposure and the population‐weighted 6mDM8h O_3_ exposure.

The residential scenario of a 50% transition from residential solid fuel use to LPG outside the GBA result in the largest reductions across China in both PM_2.5_ exposure (1.4–14.3 μg m^−3^, 3–18%) and O_3_ exposure (0.3–1.8 ppb, 0–2%). The reductions in PM_2.5_ exposure are largest in North China (14.3 μg m^−3^, 17%) and exceed 10 μg m^−3^ in South West, South Central, East, and North East China (Figure S3). The reductions in O_3_ exposure are largest in South West (1.8 ppb, 2%) and South Central China (1.0 ppb, 1%, Figure S4). The residential scenario resulted in the largest public health benefits across China. The reduction in PM_2.5_ exposure avoids 188,200 (95UI: 182,900–194,000) premature deaths (277 DALYs per 100,000, 95UI: 245–315), of which 29% are in East China and 24% are in South Central China (Figure S5). The reduction in O_3_ exposure avoids 3,200 (95UI: 2,300–4,400) premature deaths (4 DALYs per 100,000, 95UI: 2–5), of which 66% are in South Central and South West China (Figure S6). The reduction of residential solid fuel use emissions outside the GBA avoids 2,400 (95UI: 2,200–2,500) premature deaths (116 DALYs per 100,000, 95UI: 101–133) each year inside the GBA.

The next largest public health benefits result from the agricultural scenario of a 30% reduction in NH_3_ emissions from fertilizer use outside the GBA. Under the agricultural scenario, PM_2.5_ exposure decreases by 1.2–3.8 μg m^−3^ (3–5%) across China. The reductions in PM_2.5_ exposure are largest in North (3.8 μg m^−3^, 4%), South Central (3.7 μg m^−3^, 5%), and South West China (3.5 μg m^−3^, 5%). However, the agricultural scenario increases O_3_ exposure by 0.1–0.7 ppb (0–1%), mainly across North and South West China (both 0.7 ppb, 1%). Despite this increase in O_3_ exposure, there are 56,500 (95UI: 55,500–57,600) fewer premature deaths (98 DALYs per 100,000, 95UI: 87–111) across China, as the O_3_ disease burden is ∼5% of the PM_2.5_ disease burden. The reduction of agricultural fertilizer emissions outside the GBA avoids 1,900 (95UI: 1,800–1,900) premature deaths (93 DALYs per 100,000, 95UI: 82–105) each year inside the GBA.

The regional land transport scenario of an 80% reduction in NO_X_ emissions across China reduces PM_2.5_ exposure by 0.3–2.8 μg m^−3^ (1–4%) across China. The reductions in PM_2.5_ exposure are largest in South Central and South West China (both 2.8 μg m^−3^, 4%). However, under the regional land transport scenario, O_3_ exposure increases by 1.4 ppb (2%) across China. O_3_ exposure increases in East, North, South Central, and North East China (0.6–3.5 ppb, 1–6%), while O_3_ exposure decreases in South West and North West China (1.0–1.6 ppb, 2%). Reducing NO_*X*_ emissions decreases O_3_ exposure in the rural areas of South West China, as they are considered to be NO_X_−limited (Jin & Holloway, 2015; Wang et al., 2017). In contrast, reducing NO_X_ emissions increases O_3_ exposure in the urban areas of East China, as they are considered to be VOC−limited (Jin & Holloway, 2015; Wang et al., 2017). Despite the dichotomy in exposure change, there are 27,200 (95UI: 24,200–29,900) fewer premature deaths (72 DALYs per 100,000, 95UI: 62–84) across China, as the decrease in the PM_2.5_ disease burden outweighs the increase in the O_3_ disease burden. The regional reduction in land transport NO_*X*_ emissions avoids 200 (95UI: 100–300) premature deaths (17 DALYs per 100,000, 95UI: 17–19) each year inside the GBA.

The regional industrial scenario of a 10% reduction in VOC emissions across China reduces both PM_2.5_ exposure (0.4–1.1 μg m^−3^, 1%) and O_3_ exposure (0.3–0.9 ppb, 0–1%) across China. The largest reduction in both PM_2.5_ and O_3_ exposure are in East, North, and South Central China. Reducing VOC emissions in urban areas across China reduces O_3_ concentrations, as these areas are considered to be VOC‐limited (Jin & Holloway, 2015; Wang et al., 2017). These exposure reductions avoid 14,300 (95UI: 13,900–14,800) premature deaths (18 DALYs per 100,000, 95UI: 16–20) from PM_2.5_ exposure and avoid 2,800 (95UI: 2,000–4,400) premature deaths (1 DALYs per 100,000, 95UI: 1–2) from O_3_ exposure. Within these public health benefits, there are 900 (95UI: 900–1,000) fewer premature deaths (42 DALYs per 100,000, 95UI: 35–48) each year inside the GBA.

The local industrial and land transport scenarios to the GBA produce minor changes in both PM_2.5_ and O_3_ exposure.

## 4 Discussion

Our scenarios inside the GBA focused on industrial VOC emissions and land transport NO_*X*_ emissions, as these are key local sources of precursor emissions to O_3_ concentrations where existing policies are being implemented. Our scenarios outside the GBA focused on residential emissions and agricultural NH_3_ emissions, as these key regional sources lack effective policies except in specific regions. A comparative air quality and health impact assessment of these scenarios across different regions in China enabled the impacts from emission changes inside the GBA to be contrasted against emission changes outside the GBA.

The importance of pollution transport into the GBA in winter was also seen by Hou et al. (2019), who found that pollution outside the PRD contributed 53% of the annual‐mean ambient PM_2.5_ concentrations inside the PRD, with contributions highest in winter. The larger contribution of pollution transport in winter is due to the strong prevailing North Easterly winds, which bring in pollution from the highly polluted regions outside the GBA (Hou et al., 2019).

Our work further confirms the large contribution of residential solid fuels to particulate air quality across China and suggests policies to reduce emissions from this sector would reduce PM_2.5_ and O_3_ exposure. Previous policies that promoted residential solid fuel use had large impacts on air quality and public health in China. For example, the China's Huai River policy provided heavily subsidized coal for household heating to the north of the Huai River but not to the south from 1950 to 1980, and resulted in ambient PM_10_ exposure increasing by 41.7 μg m^−3^ in the north which reduced life expectancy by 3.1 years (Ebenstein et al., 2017; Yuyu Chen et al., 2013). Residential solid fuel use emissions from cooking did decrease from 1992 to 2015 in China (Aunan & Wang, 2014; Du et al., 2018; S. Tao et al., 2018; Yilin Chen et al., 2016), leading to large public health benefits (Zhao et al., 2018; H. Zheng et al., 2019). However, these improvements were primarily due to urbanization and increased incomes rather than specific policies (Zhao et al., 2018). New policies focusing on residential solid fuels are required because residential solid fuels are still widely used for heating (S. Tao et al., 2018; Zhu et al., 2018), urbanization and income growth are projected to slow (L. Jiang & O'Neill, 2017), and stacking clean fuels with solid fuels is still persistent (Barrington‐Leigh et al., 2019; Zhu et al., 2018).

The 2018–2020 3−year plan introduced the first specific policies for residential heating in Beijing−Tianjin−Hebei and the surrounding areas. This policy is estimated to reduce PM_2.5_ emissions from household fuels by 15%–41%, reduce total PM_2.5_ exposure by 10–44%, and avoid 32,000–56,000 premature deaths (J. Liu et al., 2019a; Meng et al., 2019; Qin et al., 2017; Zhao et al., 2018). This exposure change is of a similar magnitude to our 17% reduction in PM_2.5_ exposure in North China under the residential scenario, where 28,900 (95UI: 28,100–29,800) premature deaths were avoided. There are currently no specific policies for tackling residential cooking and heating from solid fuels in South China, despite this source making a large contribution to current emissions. For example, solid fuels account for 80% of the total fuel consumption in terms of energy generated within rural households in the PRD and are widely used in South West China (X. Jiang et al., 2015; S. Tao et al., 2018; Zhu et al., 2018). Our residential scenario reduced PM_2.5_ exposure by 10.3 μg m^−3^ in South Central China and by 11.9 μg m^−3^ in South West China, avoiding 73,800 (95UI: 71,700–76,200) premature deaths across these regions. We found that air quality inside the GBA can be improved from reductions in residential emissions outside the GBA, as pollution transport into the GBA is important. These reductions in residential emissions may be realized in North China from current policies, however, solid fuel use remains high in many rural areas of South China (Yun et al., 2020), where there are currently no specific policies to reduce residential emissions.

Reducing residential solid fuel use will also lead to further public health benefits from reductions in household PM_2.5_ exposure (Aunan et al., 2018; Chan et al., 2019; Kim et al., 2016; Li et al., 2019b; S. Li et al., 2019d; Vermeulen et al., 2019; K. Yu et al., 2018; Zhao et al., 2018). A reduction in residential solid fuel use may also reduce greenhouse gas emissions (Rive & Aunan, 2010; Smith & Haigler, 2008), depending on the replacement fuel (Qin et al., 2017; Yang & Jackson, 2013). The transition from residential solid fuel use to LPG may change VOC emissions and therefore O_3_ formation, though these changes are not accounted for here (Conibear et al., 2020; Lyu et al., 2017). The reduction in agricultural NH_3_ emissions could increase acid rain (M. Liu et al., 2019b), and may therefore be more suitable for areas with little acid rain and large PM_2.5_ concentrations rather than areas with existing acid rain issues.

We found that reducing NO_X_ emissions from land transport by 80% across China avoided 33,000 (95UI: 32,100–34,000) premature deaths from PM_2.5_ exposure. Anenberg et al., (2017) estimated that the China 6 emission standard would avoid 69,500 premature deaths from PM_2.5_ exposure. These larger estimates from Anenberg et al. (2017) were for 2040 and included increased car ownership, population growth, and aging. A transition away from diesel, such as the China six emission standard, will also likely lead to reductions in primary particulate emissions from land transport, that are not accounted for here.

The GBA aims that by 2025 there will be a further 30% reduction in annual‐mean ambient PM_2.5_ concentrations to reach 25 μg m^−3^, and that daily‐maximum 8‐hour O_3_ concentrations are under 75 ppb (Hong Kong Environment Bureau & Ministry of Environmental Protection of China, 2019; Tsinghua University, 2017; United Nations Environment Programme, 2019). If we adjust our simulated PM_2.5_ exposure in the GBA by the positive bias (13%), then our control PM_2.5_ exposure is just under the annual standard (35 μg m^−3^) at 34 μg m^−3^. If we adjust our simulated O_3_ exposure in the GBA by the negative bias (25%), then our control O_3_ (6mDM8h) exposure is 77 ppb. Using these bias−adjusted exposures for the different scenarios, we find that no single scenario achieves either of these 2025 goals in the GBA. Furthermore, although the residential scenario provided large public health benefits by avoiding 191,400 (95UI: 185,200–198,400) premature deaths each year across China, this represents only 7% of the current disease burden from ambient air pollution exposure. This means that even under this residential scenario, more than 2,500,000 premature deaths would still occur every year due to ambient air pollution exposure. This highlights that further strong emission reductions are required in order to reach air quality standards that protect public health.

In recent years, there have been substantial reductions in PM_2.5_ concentrations but increasing O_3_ concentrations across China (Silver et al., 2018, 2020b). This highlights the need to identify policies that reduce both PM_2.5_ and O_3_ exposure. We found that the residential and regional industrial scenarios achieved reductions in both PM_2.5_ and O_3_ exposure, whereas the agricultural and regional land transport scenarios decreased PM_2.5_ exposure at the expense of increased O_3_ exposure. Reduced NO_*X*_ emissions can increase O_3_ concentrations in urban regions due to less nitric oxide plus O_3_, and reduce PM_2.5_ concentrations by reducing nitrate aerosol concentrations. K. Li et al. (2019c, 2020) suggested that recent increases in O_3_ concentrations were attributable to decreased NO_X_ emissions in VOC−limited urban areas and to decreased PM_2.5_ concentrations that have slowed aerosol radical sinks.

Emissions over China are uncertain, and future work reducing these uncertainties will improve the accuracy of comparative health impact assessments of different scenarios (M. Li et al., 2017; Saikawa et al., 2017). The residential scenario applied reductions across the whole sector, rather than by specific solid fuel type, due to limited input data availability. The impacts of this constraint will be small as the majority of residential emissions are from solid fuel use (Carter et al., 2019; S. Tao et al., 2018). However, the accuracy of this scenario would be improved by the availability of fuel specific emission inventories with time‐activity data. The future availability of higher resolution emissions and baseline health data may improve the accuracy of this health impact assessment. Models with higher spatial resolution may better capture the spatial and temporal variation of PM_2.5_ concentrations (Y. Li et al., 2016; Punger & West, 2013; Thompson et al., 2014). Simulated O_3_ concentrations may be less sensitive to increases in spatial resolution, relative to PM_2.5_ concentrations (Punger & West, 2013; Thompson & Selin, 2012; Wild & Prather, 2006).

Currently, there are three main types of RR model for health impact assessments of PM_2.5_ exposure: log‐linear (Pope III et al., 2002), integrated−exposure response (IER, R. Burnett et al., 2014), and the GEMM (Burnett et al., 2018). The IER and GEMM are both supra‐linear models with steeper increases in RR at lower exposures which flatten off at higher exposures, while the log−linear models the logarithm of the RR as linear to the exposure change. The GEMM model has a larger increase in the RR at lower exposures (<30 μg m^−3^) compared to the log‐linear model (Burnett & Cohen, 2020). However, the log‐linear model has substantially larger increases in the RR at higher exposures (>50 μg m^−3^), which are considered biologically implausible and unsuitable for health impact assessments over areas with high PM_2.5_ exposures (Burnett & Cohen, 2020). The IER risks are smaller than the GEMM risks due to the inclusion of epidemiological data from second−hand smoking and household air pollution in the IER, and because the GEMM model includes more causes of deaths than the six causes included in the IER (Burnett & Cohen, 2020). Hence, for single national assessments with high PM_2.5_ exposures the GEMM model is a suitable choice of exposure‐outcome association, though the disease burden estimates will be larger than those obtained from global comparative assessments using the IER. The differences between these RR models will be reduced when further data is included from epidemiological studies over areas with high PM_2.5_ exposure (Pope III, Coleman, Pond, & Burnett, 2019), such as the China cohort (Yin et al., 2017).

Across the whole O_3_ exposure range (35–200 ppb), the relationship between O_3_ exposure and PAF is non−linear (Equation 5, Conibear et al., 2020; GBD 2017 Risk Factor Collaborators, 2018). However, for O_3_ concentrations of 50–80 ppb, the association between O_3_ exposure and PAF is approximately linear. Most of the population in China is exposed to 6mDM8h O_3_ exposures in this range, and hence, the relative impacts of O_3_ exposure on disease burden are approximately proportionally linear (Figure 5).

In this study, we focused on the impacts of individual realistic air quality policy scenarios on ambient air pollution and human health. The impacts of these scenarios are policy‐specific and do not represent general attributions to air quality exposure, where residential and industrial emissions have been found to dominate across China (Reddington et al., 2019). In reality, multiple policies will overlap and interact, and future work is needed to study these combined impacts.

## 5 Conclusion

In this study, we used a regional chemical transport model to explore the impacts of different emission scenarios on ambient air pollution exposure and public health across China. Our study focused on the GBA in South China, which suffers from poor air quality.

We find that in winter and autumn, emission sources outside the GBA contribute more to PM_2.5_ exposure in the GBA than emission sources inside the GBA. PM_2.5_ exposure in the GBA reduced by 8% in winter and 6% in autumn when emissions were reduced by 15% outside the GBA, larger than when emissions were reduced by 15% inside the GBA (4% in winter and 5% in autumn).

We quantified the potential impacts of six different air quality policy scenarios on ambient air pollution exposure and public health across China. Our selected policies addressed the land transport and industrial sectors where there are existing policies, as well as the residential and agricultural sectors where policy controls are less developed.

The scenario of a 50% transition from residential solid fuel use to LPG outside the GBA had the greatest benefit on public health across China and inside the GBA. PM_2.5_ exposure reduced by 10.6 μg m^−3^ (15%) and O_3_ exposure reduced by 0.8 ppb (1%) across China, with large reductions in North, South West, South Central, East, and North East China. The residential scenario avoided 191,400 (95UI: 185,200–198,400) premature deaths each year across China, with 2,400 (95UI: 2,200–2,500) fewer premature deaths inside the GBA.

The next largest public health benefit came from the agricultural scenario of a 30% reduction in NH_3_ emissions from fertilizer use outside the GBA. The agricultural scenario reduced PM_2.5_ exposure by 3.2 μg m^−3^ (4%) across China, with the largest reductions in North, South Central, and South West China. However, O_3_ exposure increased by 0.5 ppb (1%) across China, mainly across North and South West China. As the O_3_ disease burden is approximately 5% of the PM_2.5_ disease burden, the agricultural scenario still had a large net benefit to public health with 56,500 (95UI: 55,500–57,600) avoided premature deaths across China, of which 1,900 (95UI: 1,800–1,900) were inside the GBA.

The 2018–2020 3‐year plan requires the PRD to enhance land transport standards and transition away from diesel to decrease NO_X_ emissions. We simulated an approximation to this transition by reducing land transport NO_*X*_ emissions by 80%. If this policy is applied only within the GBA, our simulations suggest PM_2.5_ and O_3_ exposure remain relatively unchanged. If this policy is applied nationwide, PM_2.5_ exposure decreased by 1.9 μg m^−3^ (3%) across China, primarily from reductions in South Central and South West China. However, O_3_ exposure increased by 1.4 ppb (2%) across China, where exposure increased in East, North, South Central, and North East China, and decreased in South West and North West China. This scenario resulted in 27,200 (95UI: 24,200–29,900) fewer premature deaths each year, of which 200 (95UI: 100–300) avoided premature deaths were inside the GBA.

The 2018–2020 3‐year plan aims to tackle the recent rise in VOC emissions by requiring a 10% emission reduction in industrial VOC emissions. Our simulations suggest that if this policy is applied solely within the GBA, then there are only minor changes in PM_2.5_ and O_3_ exposure. If this policy is applied nationwide, then PM_2.5_ exposure reduced by 0.9 μg m^−3^ (1%) and O_3_ exposure reduced by 0.7 ppb (1%), both primarily due to reductions in East, North, and South Central China. The regional industrial scenario avoided 17,100 (95UI: 15,900–19,200) premature deaths across China, of which 900 (95UI: 900–1,000) avoided premature deaths were inside the GBA.

Overall, we find that controls on residential solid fuel and agricultural emissions would provide the largest public health benefits both across China and inside the GBA. There are currently no specific policies for reducing residential solid fuel use or agricultural emissions in South China, highlighting a major opportunity for targeted emission controls. Improving air quality inside the GBA will require coordinated regional emission reduction policies both inside and outside the GBA from key contributing sectors.

## Conflict of Interest

The authors declare no conflicts of interest relevant to this study.

## Supporting information

Supporting Information S1

## Data Availability

The air pollution and health impact assessment data per Chinese province and GBA prefecture that support the findings of this study are available at doi.org/10.5518/919. Code to setup and run WRFChem (using WRFotron version 2.0) is available through Conibear and Knote (2020).
