# Supplementary material for: Regional Policies Targeting Residential Solid Fuel and Agricultural Emissions Can Improve Air Quality and Public Health in the Greater Bay Area and Across China
Source: Geohealth. 2021 Apr 1;5(4):e2020GH000341. doi: 10.1029/2020GH000341 (PMC8057822; doi:10.1029/2020GH000341)
Supplement: Supplementary file 1 — Supporting Information S1 [file GH2-5-e2020GH000341-s001.pdf]

AGU PUBLICATIONS

GeoHealth

Supporting information for

Regional policies targeting residential solid fuel and agricultural emissions can improve air quality and public health in the Greater Bay Area and across China

Luke Conibear*[,1], Carly L. Reddington[1], Ben J. Silver[1], Christoph Knote[2], Stephen R. Arnold[1], and Dominick V. Spracklen[1]

[1] Institute for Climate and Atmospheric Science, School of Earth and Environment, University of Leeds, Leeds, UK

[2] Faculty of Medicine, University of Augsburg, Germany

* Corresponding author: Luke Conibear (L.A.Conibear@leeds.ac.uk)

Contents of this file

Supplementary Table 1: Model setup used in the Weather Research and Forecasting model online–coupled with Chemistry (WRFChem) simulations.

Supplementary Table 2: Global Exposure Mortality Model (GEMM) fit parameters for the health impact assessment from ambient fine particulate matter ($PM_{2.5}$) exposure (Burnett et al., 2018). All−regions, including China cohort, non−accidental function (non−communicable disease plus lower respiratory infections).

Supplementary Table 3: The impacts of policy scenarios on air quality and public health per region in China (see Supplementary Figure 2 for more details). Air quality represented by population−weighted annual−mean ambient fine particulate matter ($PM_{2.5}$) exposure and population−weighted maximum 6−monthly−mean daily−maximum 8−hour ambient ozone (O_3) exposure (6mDM8h). Disease burden estimates for premature mortalities (MORT, annual−sum, rounded to the nearest 100) and rate of disability−adjusted life years (DALYs, annual−mean) per 100,000 population. Scenarios are for the control (CTL) and the residential (RES), industrial within the Guangdong−Hong Kong−Macau Greater Bay Area (GBA, IND−GBA), industrial over China (IND−CHN), land transport within the GBA (TRA−GBA), land transport over China (TRA−CHN), and agriculture (AGR) scenarios relative to the control. Values in parentheses represent the 95% uncertainty intervals.

Supplementary Figure 1: Domains for Weather Research and Forecasting model online–coupled with Chemistry (WRFChem) simulations

Supplementary Figure 2: Regional groupings for (a) North China (Beijing, Tianjin, Hebei, Shanxi, and Inner Mongolia), North East China (Liaoning, Jilin, and Heilongjiang), East China (Shanghai, Jiangsu, Zhejiang, Anhui, Fujian, Jiangxi, and Shandong), South Central China (Henan, Hubei, Hunan, Guangdong, Guangxi, Hainan, Hong Kong, and Macau) including the Guangdong−Hong Kong−Macau Greater Bay Area (GBA), South West China (Chongqing, Sichuan, Guizhou, Yunnan, and Tibet), North West China (Shaanxi, Gansu, Qinghai, Ningxia, and Xinjiang), and (b) the GBA individually.

Supplementary Figure 3: Change in the annual−mean ambient fine particulate matter ($PM_{2.5}$) concentrations in China relative to the control (CTL) for each scenario; (a) residential (RES), (b) industry for China (IND−CHN), (c) land transport for China (TRA−CHN), and (d) agriculture (AGR).

Supplementary Figure 4: Change in the annual−mean ambient ozone (O_3) concentrations in China relative to the control (CTL) for each scenario; (a) residential (RES), (b) industry for China (IND−CHN), (c) land transport for China (TRA−CHN), and (d) agriculture (AGR).

Supplementary Figure 5: Change in the rate of disability−adjusted life years (DALYs) per 100,000 population from ambient fine particulate matter ($PM_{2.5}$) exposure in China relative to the control (CTL) for each scenario; (a)

residential (RES), (b) industry for China (IND−CHN), (c) land transport for China (TRA−CHN), and (d) agriculture (AGR).

Supplementary Figure 6: Change in the rate of disability−adjusted life years (DALYs) per 100,000 population from ambient ozone (O_3) exposure in China relative to the control (CTL) for each scenario; (a) residential (RES), (b) industry for China (IND−CHN), (c) land transport for China (TRA−CHN), and (d) agriculture (AGR).

Additional Supporting Information (Files uploaded separately)

The air pollution and health impact assessment data per Chinese province and GBA prefecture that support the findings of this study are available at doi.org/10.5518/919.

Supplementary Table 1: Model setup used in the Weather Research and Forecasting model online–coupled with Chemistry (WRFChem) simulations.

**Model Setup and Parameterisation**	
**Process**	**Method**
**Timestep**	180 seconds.
**Horizontal**	Parent grid on a resolution of 30 km along a 170 × 170 Lambert conformal conical grid, with a 10 km nest over Guangdong–Hong Kong–Macau Greater Bay Area (GBA).
**Vertical**	33 vertical levels, with 38 meteorological levels.
**Microphysics**	Morrison two–moment scheme (Morrison, Thompson, & Tatarskii, 2009).
**Radiation**	Rapid radiative transfer model for general circulation models (RRTMG), short–wave and long–wave (Iacono et al., 2008).
**Boundary layer physics**	Mellor–Yamada Nakanishi and Niino 2.5 (Nakanishi & Niino, 2006).
**Land surface**	Noah Land Surface Model (Ek et al., 2003).
**Convection**	Grell 3–D ensemble (Grell & Devenyi, 2002).
**Gas–phase chemistry**	Extended Model for Ozone and Related Chemical Tracers (MOZART, Emmons et al., 2010; A. Hodzic & Jimenez, 2011; Knote et al., 2014).
**Aerosol**	Updated Model for Simulating Aerosol Interactions and Chemistry (MOSAIC) with aqueous chemistry, volatility basis set secondary organic aerosol production, and 4 sectional bins (Alma Hodzic & Knote, 2014; Knote, Hodzic, & Jimenez, 2015; Zaveri, Easter, Fast, & Peters, 2008).
**Photolysis**	Updated tropospheric ultraviolet-visible (TUV) photolysis based originally on Tie et al., (2003).
**Dust**	Global Ozone Chemistry Aerosol Radiation and Transport (GOCART) with Air Force Weather Agency (AFWA) modifications (Legrand et al., 2019).
**Initial & boundary chemistry**	MOZART / Goddard Earth Observing System (GEOS) Model (National Center for Atmospheric Research, 2016).
**Initial & boundary meteorology**	European Centre for Medium–Range Weather Forecasts (ECMWF) global reanalysis products (Dee et al., 2011).

Supplementary Table 2: Global Exposure Mortality Model (GEMM) fit parameters for the health impact assessment from ambient fine particulate matter ($PM_{2.5}$) exposure (Burnett et al., 2018). All−regions, including China cohort, non−accidental function (non−communicable disease plus lower respiratory infections).

Age group	θ	Standard error in θ	α	μ	υ
**25+**	0.1430	0.01807	1.6	15.5	36.8
**25−29**	0.1585	0.01477	1.6	15.5	36.8
**30−35**	0.1577	0.01470	1.6	15.5	36.8
**35−39**	0.1570	0.01463	1.6	15.5	36.8
**40−44**	0.1558	0.01450	1.6	15.5	36.8
**45−49**	0.1532	0.01425	1.6	15.5	36.8
**50−54**	0.1499	0.01394	1.6	15.5	36.8
**55−59**	0.1462	0.01361	1.6	15.5	36.8
**60−64**	0.1421	0.01325	1.6	15.5	36.8
**65−69**	0.1374	0.01284	1.6	15.5	36.8
**70−74**	0.1319	0.01234	1.6	15.5	36.8
**75−79**	0.1253	0.01174	1.6	15.5	36.8
**80+**	0.1141	0.01071	1.6	15.5	36.8

Supplementary Table 3: The impacts of policy scenarios on air quality and public health per region in China (see Supplementary Figure 2 for more details). Air quality represented by population−weighted annual−mean ambient fine particulate matter ($PM_{2.5}$) exposure and population−weighted maximum 6−monthly−mean daily−maximum 8−hour ambient ozone (O_3) exposure (6mDM8h). Disease burden estimates for premature mortalities (MORT, annual−sum, rounded to the nearest 100) and rate of disability−adjusted life years (DALYs, annual−mean) per 100,000 population. Scenarios are for the control (CTL) and the residential (RES), industrial within the Guangdong−Hong Kong−Macau Greater Bay Area (GBA, IND−GBA), industrial over China (IND−CHN), land transport within the GBA (TRA−GBA), land transport over China (TRA−CHN), and agriculture (AGR) scenarios relative to the control. Values in parentheses represent the 95% uncertainty intervals.

		CTL	RES	IND−GBA	IND−CHN	TRA−GBA	TRA−CHN	AGR
**$PM_{2.5}$ (μg m^{-3})**	**China**	72.8	−10.6	0.0	−0.9	0.0	−1.9	−3.2
	**GBA**	39.6	−1.4	+0.1	−0.5	+0.1	−0.3	−1.2
	**North China**	87.1	−14.3	0.0	−1.0	+0.1	−1.7	−3.8
	**North East China**	55.8	−10.0	+0.1	−0.6	0.0	−0.6	−1.9
	**East China**	74.2	−10.2	0.0	−1.1	0.0	−1.1	−3.1
	**South Central China**	77.6	−10.3	0.0	−0.9	0.0	−2.8	−3.7
	**South West China**	71.5	−11.9	+0.1	−0.7	+0.1	−2.8	−3.5
	**North West China**	44.7	−5.3	0.0	−0.4	0.0	−1.5	−2.1
**O_3 6mDM8h (ppb)**	**China**	63.5	−0.8	0.0	−0.7	0.0	+1.4	+0.5
	**GBA**	61.3	−0.7	0.0	−0.6	0.0	+1.0	+0.1
	**North China**	61.2	−0.4	0.0	−0.7	0.0	+3.2	+0.7
	**North East China**	54.8	−0.3	0.0	−0.5	0.0	+0.6	+0.4
	**East China**	60.4	−0.6	0.0	−0.9	0.0	+3.5	+0.5
	**South Central China**	65.7	−1.0	0.0	−0.7	0.0	+0.7	+0.4
	**South West China**	72.9	−1.8	0.0	−0.6	0.0	−1.6	+0.7
	**North West China**	63.7	−0.6	0.0	−0.3	0.0	−1.0	+0.4
**$PM_{2.5}$ MORT (yr^{-1})**	**China**	2,778,700 (2,700,700 to	−188,200 (−182,900 to	+800 (+800 to	−14,300 (−13,900 to	+1,000 (+1,000 to	−33,000 (−32,100 to	−58,600 (−57,000 to

		2,864,900)	−194,000)	+900)	−14,800)	+1,000)	−34,000)	−60,400)
	**GBA**	104,700 (101,700 to 107,900)	−2,200 (−2,100 to −2,300)	+100 (+100 to +100)	−800 (−800 to −800)	+100 (+100 to +100)	−400 (−400 to −400)	−1,900 (−1,800 to −1,900)
	**North China**	384,600 (373,800 to 396,500)	−28,900 (−28,100 to −29,800)	+100 (+100 to +100)	−1,700 (−1,600 to −1,700)	+100 (+100 to +100)	−3,400 (−3,300 to −3,500)	−8,000 (−7,800 to −8,300)
	**North East China**	195,400 (190,000 to 201,500)	−21,500 (−20,900 to −22,200)	+100 (+100 to +100)	−1,200 (−1,100 to −1,200)	+100 (+100 to +100)	−1,300 (−1,200 to −1,300)	−3,800 (−3,700 to −4,000)
	**East China**	851,800 (827,900 to 878,200)	−54,000 (−52,500 to −55,700)	+300 (+300 to +300)	−5,500 (−5,300 to −5,700)	+300 (+200 to +300)	−5,500 (−5,400 to −5,700)	−17,200 (−16,700 to −17,700)
	**South Central China**	805,000 (782,400 to 830,000)	−44,400 (−43,100 to −45,800)	+200 (+200 to +200)	−3,800 (−3,700 to −3,900)	+300 (+300 to +300)	−12,200 (−11,800 to −12,600)	−16,800 (−16,300 to −17,300)
	**South West China**	387,900 (377,000 to 399,900)	−29,400 (−28,600 to −30,400)	+200 (+200 to +200)	−1,500 (−1,500 to −1,600)	+200 (+200 to +200)	−7,700 (−7,400 to −7,900)	−8,800 (−8,600 to −9,100)
	**North West China**	154,000 (149,700 to 158,800)	−10,000 (−9,700 to −10,300)	0 (0 to 0)	−600 (−600 to −700)	0 (0 to 0)	−2,900 (−2,800 to −3,000)	−4,000 (−3,800 to −4,100)
**$PM_{2.5}$ DALYs rate (per 100,000 yr^{-1})**	**China**	4,476 (3,947 to 5,084)	−277 (−245 to −315)	+1 (+1 to +1)	−18 (−16 to −20)	+1 (+1 to +2)	−66 (−58 to −75)	−99 (−88 to −113)
	**GBA**	4,887 (4,309 to 5,551)	−111 (−98 to −126)	+5 (+4 to +5)	−38 (−33 to −43)	+5 (+5 to +6)	−24 (−21 to −28)	−93 (−82 to −106)
	**North China**	6,314 (5,569 to 7,170)	−469 (−414 to −532)	0 (0 to 0)	−28 (−25 to −32)	+2 (+2 to +2)	−62 (−54 to −70)	−164 (−145 to −186)
	**North East China**	4,900 (4,321 to 5,566)	−526 (−464 to −598)	+1 (+1 to +1)	−31 (−27 to −35)	+1 (+1 to +1)	−35 (−31 to −40)	−117 (−103 to −133)
	**East China**	6,400 (5,645 to	−391 (−346 to	+3 (+3 to	−43 (−38 to	+2 (+2 to	−49 (−44 to	−151 (−133 to

		7,268)	−444)	+4)	−49)	+3)	−56)	−172)
	**South Central China**	6,358 (5,608 to 7,221)	−379 (−335 to −430)	+1 (+1 to +2)	−30 (−26 to −34)	+2 (+2 to +3)	−121 (−107 to −138)	−161 (−142 to −183)
	**South West China**	5,062 (4,464 to 5,749)	−348 (−308 to −396)	+2 (+1 to +2)	−15 (−14 to −18)	+2 (+2 to +3)	−121 (−107 to −138)	−126 (−111 to −143)
	**North West China**	3,883 (3,424 to 4,411)	−193 (−170 to −219)	0 (0 to 0)	−13 (−12 to −15)	0 (0 to 0)	−73 (−64 to −83)	−97 (−86 to −110)
**O_3 MORT (yr^{-1})**	**China**	122,800 (85,800 to 171,400)	−3,200 (−2,300 to −4,400)	0 (0 to 0)	−2,800 (−2,000 to −4,400)	0 (0 to 0)	+5,800 (+4,100 to +7,900)	+2,100 (+1,500 to +2,800)
	**GBA**	5,700 (4,000 to 7,900)	−200 (−100 to −200)	0 (0 to 0)	−100 (−100 to −200)	0 (0 to 0)	+200 (+100 to +300)	0 (0 to 0)
	**North China**	14,500 (10,100 to 20,300)	−200 (−100 to −300)	0 (0 to 0)	−400 (−300 to −500)	0 (0 to 0)	+1,700 (+1,200 to +2,300)	+400 (+300 to +500)
	**North East China**	7,000 (4,800 to 9,800)	−100 (−100 to −100)	0 (0 to 0)	−200 (−100 to −200)	0 (0 to 0)	+200 (+100 to +200)	+100 (+100 to +200)
	**East China**	32,500 (22,700 to 45,500)	−700 (−500 to −1,000)	0 (0 to 0)	−1,100 (−800 to −1,500)	0 (0 to 0)	+4,400 (+3,100 to +6,000)	+600 (+400 to +800)
	**South Central China**	37,200 (26,000 to 51,900)	−1,100 (−800 to −1,500)	0 (0 to 0)	−800 (−500 to −1,000)	0 (0 to 0)	+700 (+500 to +1,000)	+500 (+400 to +700)
	**South West China**	22,600 (15,900 to 31,400)	−1,000 (−700 to −1,300)	0 (0 to 0)	−300 (−200 to −400)	0 (0 to 0)	−900 (−700 to −1,300)	+400 (+300 to +500)
	**North West China**	9,000 (6,300 to 12,600)	−200 (−100 to −300)	0 (0 to 0)	−100 (−100 to −100)	0 (0 to 0)	−300 (−200 to −400)	+100 (+100 to +200)
**O_3 DALYs rate**	**China**	186 (122 to	−4 (−2 to	0 (0 to	−1 (−1 to	0 (0 to	−6 (−4 to	+1 (+1 to

**(per 100,000 yr^{-1})**		267)	−5)	0)	−2)	0)	−9)	+2)
	**GBA**	182 (120 to 262)	−5 (−3 to −7)	0 (0 to 0)	−4 (−2 to −5)	0 (0 to 0)	+7 (+4 to +9)	0 (0 to +1)
	**North China**	184 (120 to 263)	−2 (−1 to −3)	0 (0 to 0)	−4 (−2 to −6)	0 (0 to 0)	+15 (+10 to +21)	+4 (+2 to +5)
	**North East China**	151 (99 to 218)	−2 (−1 to −2)	0 (0 to 0)	−2 (−1 to −3)	0 (0 to 0)	0 (0 to −1)	+2 (+1 to +3)
	**East China**	186 (122 to 266)	−3 (−2 to −5)	0 (0 to 0)	−5 (−3 to −7)	0 (0 to 0)	+15 (+10 to +22)	+2 (+1 to +3)
	**South Central China**	216 (142 to 309)	−7 (−5 to −11)	0 (0 to 0)	−4 (−2 to −6)	0 (0 to 0)	−1 (0 to −2)	+2 (+1 to +3)
	**South West China**	228 (150 to 326)	−9 (−6 to −12)	0 (0 to 0)	−1 (−1 to −2)	0 (0 to 0)	−17 (−11 to −23)	+2 (+1 to +3)
	**North West China**	189 (124 to 272)	−3 (−2 to −4)	0 (0 to 0)	−1 (0 to −1)	0 (0 to 0)	−10 (−6 to −14)	+1 (0 to +2)

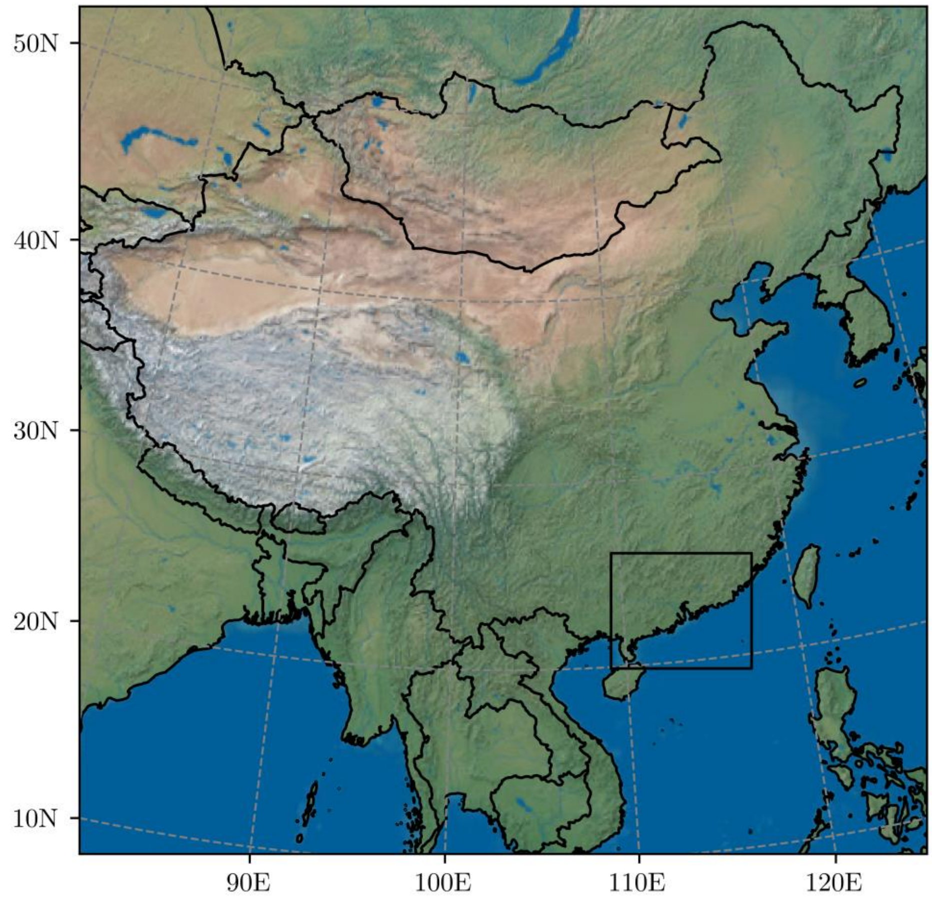

Supplementary Figure 1: Domains for Weather Research and Forecasting model online–coupled with Chemistry (WRFChem) simulations.

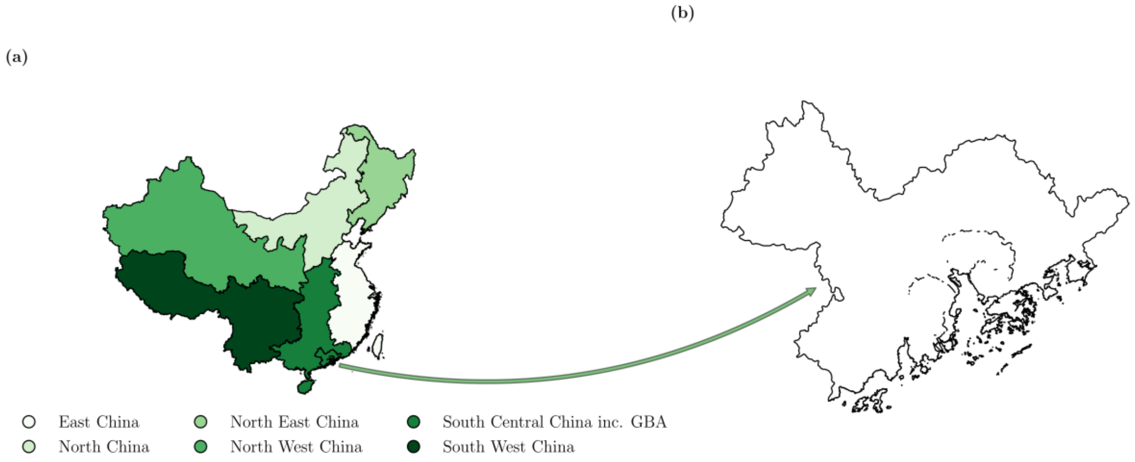

Supplementary Figure 2: Regional groupings for (a) North China (Beijing, Tianjin, Hebei, Shanxi, and Inner Mongolia), North East China (Liaoning, Jilin, and Heilongjiang), East China (Shanghai, Jiangsu, Zhejiang, Anhui, Fujian, Jiangxi, and Shandong), South Central China (Henan, Hubei, Hunan, Guangdong, Guangxi, Hainan, Hong Kong, and Macau) including the Guangdong−Hong Kong−Macau Greater Bay Area (GBA), South West China (Chongqing, Sichuan, Guizhou, Yunnan, and Tibet), North West China (Shaanxi, Gansu, Qinghai, Ningxia, and Xinjiang), and (b) the GBA individually.

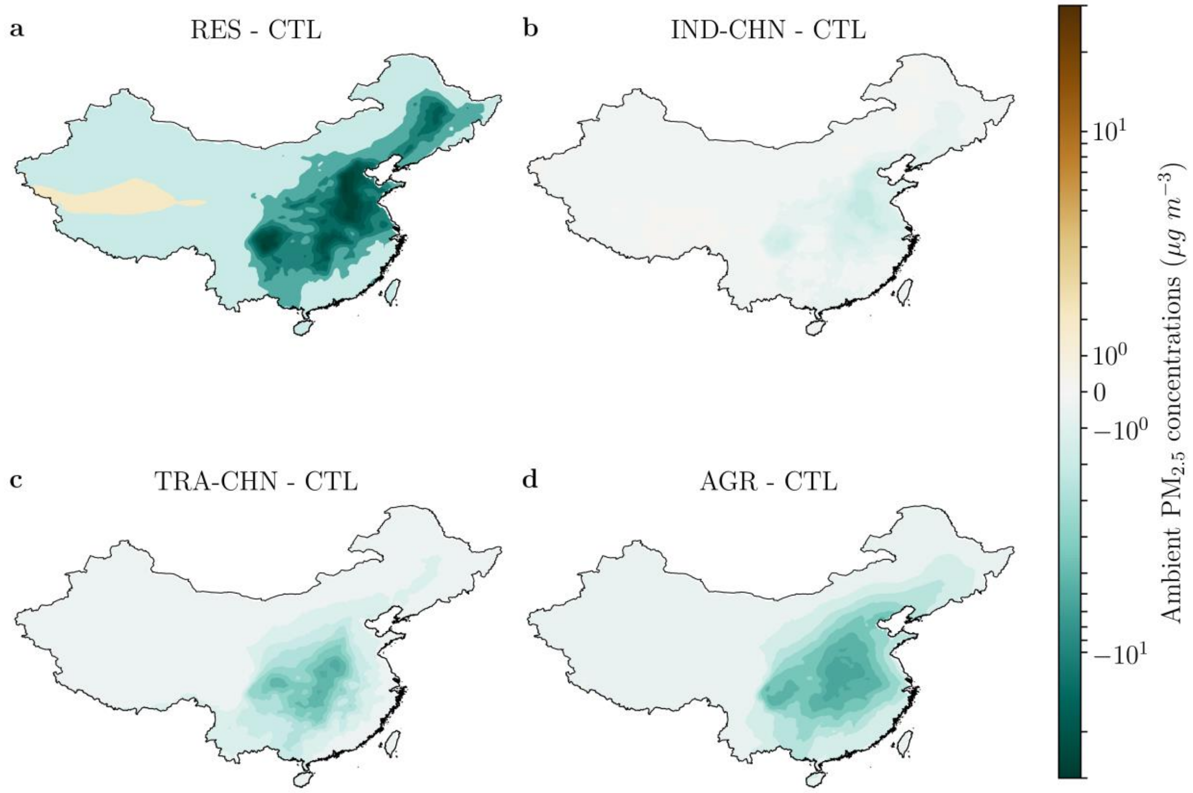

Supplementary Figure 3: Change in the annual−mean ambient fine particulate matter ($PM_{2.5}$) concentrations in China relative to the control (CTL) for each scenario; (a) residential (RES), (b) industry for China (IND−CHN), (c) land transport for China (TRA−CHN), and (d) agriculture (AGR).

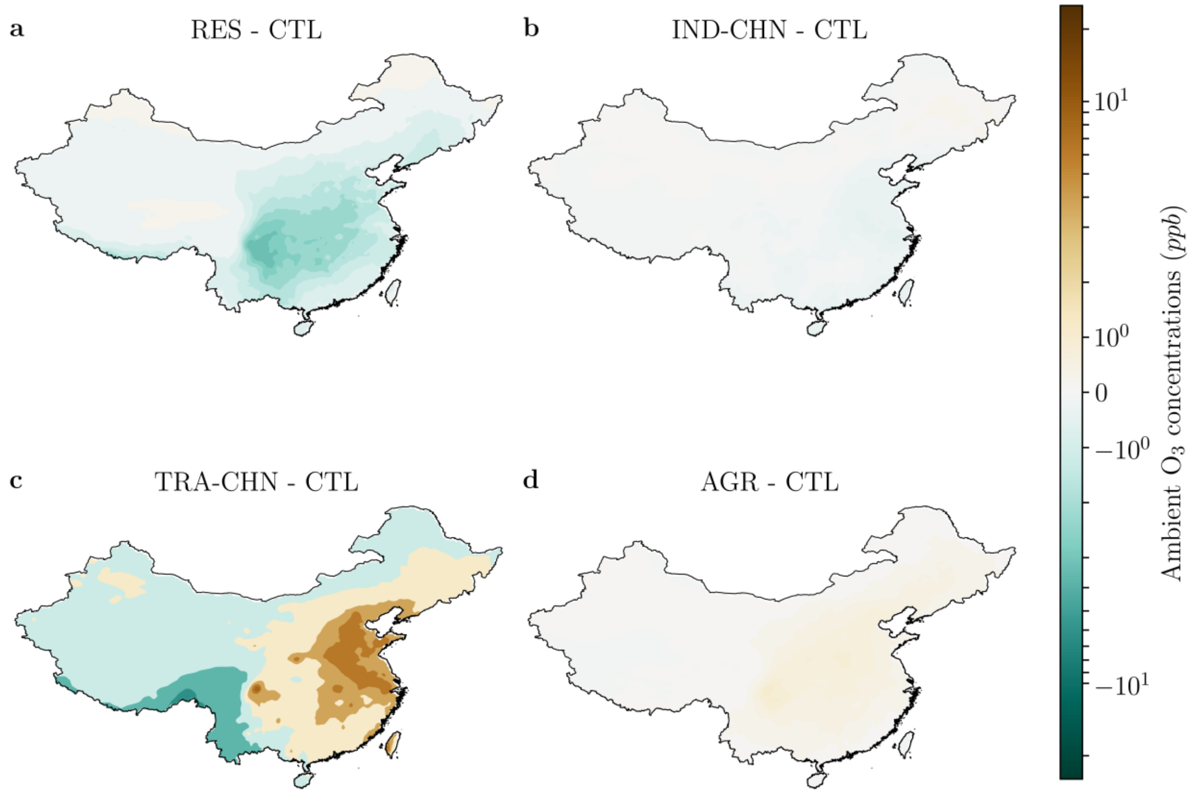

Supplementary Figure 4: Change in the annual−mean ambient ozone (O_3) concentrations in China relative to the control (CTL) for each scenario; (a) residential (RES), (b) industry for China (IND−CHN), (c) land transport for China (TRA−CHN), and (d) agriculture (AGR).

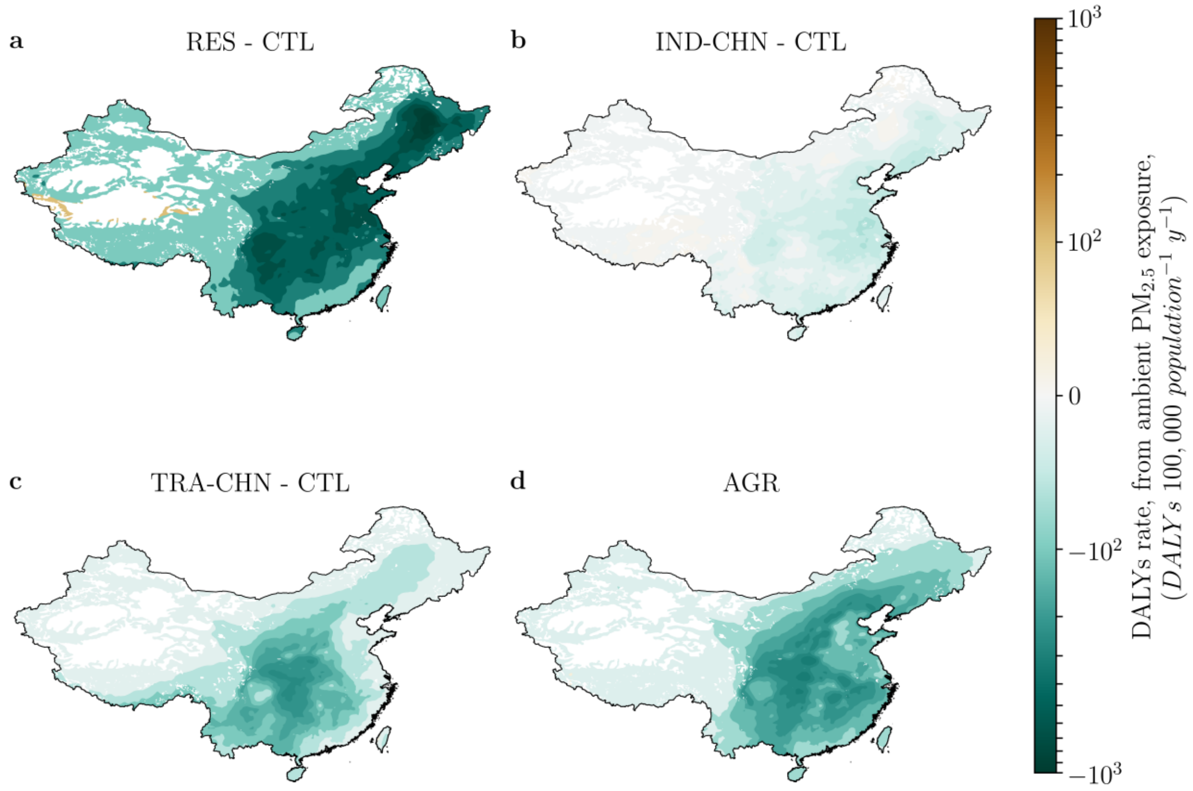

Supplementary Figure 5: Change in the rate of disability−adjusted life years (DALYs) per 100,000 population from ambient fine particulate matter ($PM_{2.5}$) exposure in China relative to the control (CTL) for each scenario; (a)

residential (RES), (b) industry for China (IND−CHN), (c) land transport for China (TRA−CHN), and (d) agriculture (AGR).

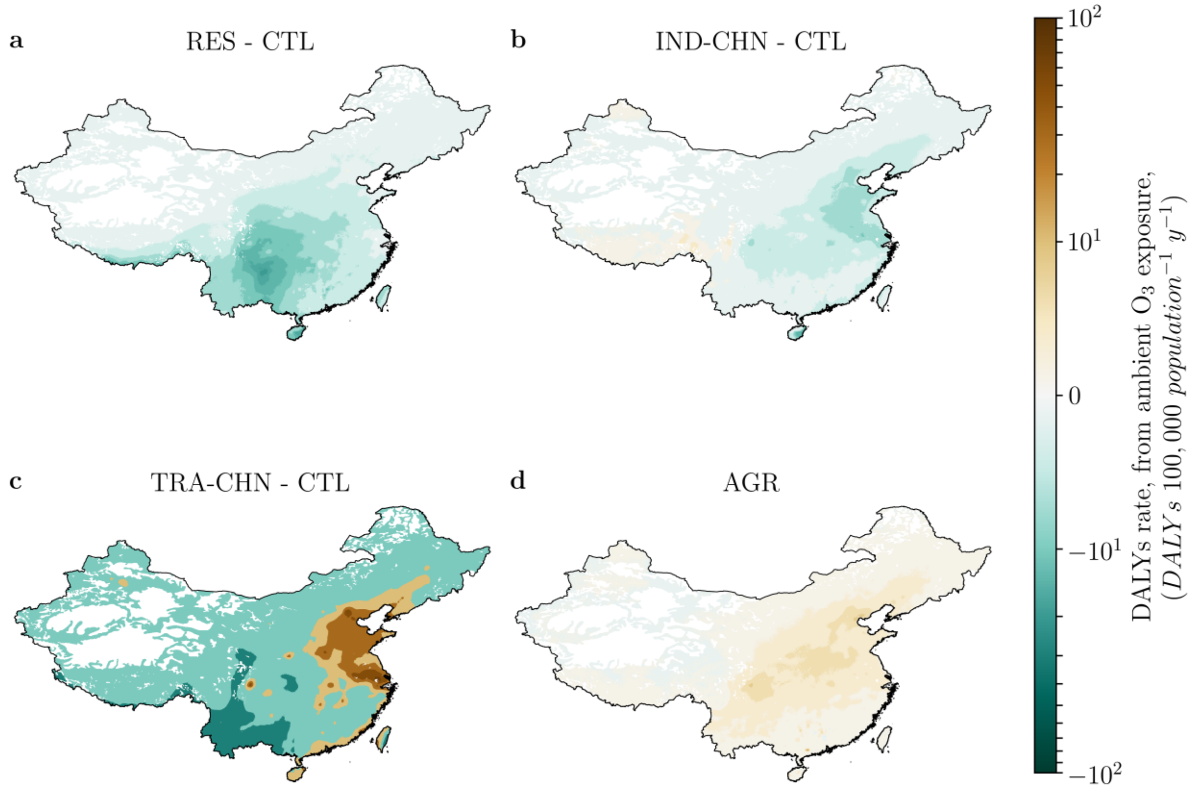

Supplementary Figure 6: Change in the rate of disability−adjusted life years (DALYs) per 100,000 population from ambient ozone (O_3) exposure in China relative to the control (CTL) for each scenario; (a) residential (RES), (b) industry for China (IND−CHN), (c) land transport for China (TRA−CHN), and (d) agriculture (AGR).

References

Burnett, R., Chen, H., Szyszkowicz, M., Fann, N., Hubbell, B., Pope, C. A., et al. (2018). Global estimates of mortality associated with long-term exposure to outdoor fine particulate matter. *Proceedings of the National Academy of Sciences*, *115*(38), 9592–9597. https://doi.org/10.1073/pnas.1803222115

Dee, D. P., Uppala, S. M., Simmons, A. J., Berrisford, P., Poli, P., Kobayashi, S., et al. (2011). The ERA-Interim reanalysis: Configuration and performance of the data assimilation system. *Quarterly Journal of the Royal Meteorological Society*, *137*(656), 553–597. https://doi.org/10.1002/qj.828

Ek, M. B., Mitchell, K. E., Lin, Y., Rogers, E., Grunmann, P., Koren, V., et al. (2003). Implementation of Noah land surface model advances in the National Centers for Environmental Prediction operational mesoscale Eta model. *Journal of Geophysical Research: Atmospheres*, *108*(D22), 8851–8867. https://doi.org/10.1029/2002JD003296

Emmons, L. K., Walters, S., Hess, P. G., Lamarque, J.-F., Pfister, G. G., Fillmore, D., et al. (2010). Description and evaluation of the Model for Ozone and Related chemical Tracers, version 4 (MOZART-4). *Geoscientific Model Development*, *3*, 43–67. https://doi.org//10.5194/gmd-3-43-2010

Grell, G. A., & Devenyi, D. (2002). A generalized approach to parameterizing convection combining ensemble and data assimilation techniques. *Geophysical Research Letters*, *29*(14), 10–13. https://doi.org/10.1029/2002GL015311

Hodzic, A., & Jimenez, J. L. (2011). Modeling anthropogenically controlled secondary organic aerosols in a megacity: a simplified framework for global and climate models. *Geoscientific Model Development*, *4*(4), 901–917. https://doi.org/10.5194/gmd-4-901-2011

Hodzic, Alma, & Knote, C. (2014). WRF-Chem 3.6.1: MOZART gas-phase chemistry with MOSAIC aerosols. *Atmospheric Chemistry Division (ACD), National Center for Atmospheric Research (NCAR)*, 7.

Iacono, M. J., Delamere, J. S., Mlawer, E. J., Shephard, M. W., Clough, S. A., & Collins, W. D. (2008). Radiative forcing by long-lived greenhouse gases: Calculations with the AER radiative transfer models. *Journal of Geophysical Research: Atmospheres*, *113*(13), 2–9. https://doi.org/10.1029/2008JD009944

Knote, C., Hodzic, A., & Jimenez, J. L. (2015). The effect of dry and wet deposition of condensable vapors on secondary organic aerosols concentrations over the continental US. *Atmospheric Chemistry and Physics*, *15*(1), 1–18. https://doi.org/10.5194/acp-15-1-2015

Knote, C., Hodzic, A., Jimenez, J. L., Volkamer, R., Orlando, J. J., Baidar, S., et al. (2014). Simulation of semi-explicit mechanisms of SOA formation from glyoxal in aerosol in a 3-D model. *Atmospheric Chemistry and Physics*, *14*(12), 6213–6239. https://doi.org/10.5194/acp-14-6213-2014

Legrand, S. L., Polashenski, C., Letcher, T. W., Creighton, G. A., Peckham, E., & Cetola, J. D. (2019). The AFWA Dust Emissions Scheme for the GOCART Aerosol Model in WRF-Chem. *Geoscientific Model Development*, *12*, 131–166. https://doi.org/10.5194/gmd-12-131-2019

Morrison, H., Thompson, G., & Tatarskii, V. (2009). Impact of Cloud Microphysics on the Development of Trailing Stratiform Precipitation in a Simulated Squall Line: Comparison of One- and Two-Moment Schemes. *Monthly Weather Review*, *137*(3), 991–1007. https://doi.org/10.1175/2008MWR2556.1

Nakanishi, M., & Niino, H. (2006). An improved Mellor-Yamada Level-3 model: Its numerical stability and application to a regional prediction of advection fog. *Boundary-Layer Meteorology*, *119*(2), 397–407. https://doi.org/10.1007/s10546-005-9030-8

National Center for Atmospheric Research. (2016). ACOM MOZART-4/GEOS-5 global model output. *UCAR*. Retrieved from http://www.acom.ucar.edu/wrf-chem/mozart.shtml

Tie, X., Madronich, S., Walters, S., Zhang, R., Rasch, P., & Collins, W. (2003). Effect of clouds on photolysis and oxidants in the troposphere. *Journal of Geophysical Research*, *108*(D20), 4642, 1–11. https://doi.org/10.1029/2003JD003659

Zaveri, R. A., Easter, R. C., Fast, J. D., & Peters, L. K. (2008). Model for Simulating Aerosol Interactions and Chemistry (MOSAIC). *Journal of Geophysical Research*, *113*(D13204), 1–29. https://doi.org/10.1029/2007JD008782
